# Magnetic Nanoparticles: Surface Effects and Properties Related to Biomedicine Applications

**DOI:** 10.3390/ijms141121266

**Published:** 2013-10-25

**Authors:** Bashar Issa, Ihab M. Obaidat, Borhan A. Albiss, Yousef Haik

**Affiliations:** 1 Department of Physics, College of Science, United Arab Emirates University, Al Ain, 15551, UAE; E-Mail: iobaidat@uaeu.ac.ae; 2 Superconductivity & Magnetic Measurements Laboratory, Physics Department, Jordan University of Science and Technology, Irbid 22110, Jordan; E-Mail: baalbiss@just.edu.jo; 3 Department of Mechanical Engineering, College of Engineering, United Arab Emirates University, Al Ain, 15551, UAE; E-Mail: yhaik@uaeu.ac.ae; 4 Centre of Research Excellence in Nanobioscience 203, Eberhart Building University of North Carolina, Greensboro, NC 27412, USA

**Keywords:** Superparamagnetism, nanoparticle, magnetic moment, exchange, anisotropy, surface spin, core-shell, ferrite, MRI, contrast agent

## Abstract

Due to finite size effects, such as the high surface-to-volume ratio and different crystal structures, magnetic nanoparticles are found to exhibit interesting and considerably different magnetic properties than those found in their corresponding bulk materials. These nanoparticles can be synthesized in several ways (e.g., chemical and physical) with controllable sizes enabling their comparison to biological organisms from cells (10–100 μm), viruses, genes, down to proteins (3–50 nm). The optimization of the nanoparticles’ size, size distribution, agglomeration, coating, and shapes along with their unique magnetic properties prompted the application of nanoparticles of this type in diverse fields. Biomedicine is one of these fields where intensive research is currently being conducted. In this review, we will discuss the magnetic properties of nanoparticles which are directly related to their applications in biomedicine. We will focus mainly on surface effects and ferrite nanoparticles, and on one diagnostic application of magnetic nanoparticles as magnetic resonance imaging contrast agents.

## 1. Introduction

The term “nanoparticles” refers to materials with at least one dimension between approximately 1 and 100 nanometers (nm) and usually contain from several hundreds to 10^5^ atoms. Magnetic materials are those materials that show a response to an applied magnetic field. They are classified into five main types; ferromagnetic, paramagnetic, diamagnetic, antiferromagnetic, and ferrimagnetic. In ferromagnetic materials (such as iron, nickel, and cobalt) an atom has a net magnetic moment due to unpaired electrons. The material is composed of domains each containing large numbers of atoms whose magnetic moments are parallel producing a net magnetic moment of the domain that points in some direction. The magnetic moments of the domains are randomly distributed giving a zero net magnetic moment of the material. When the ferromagnetic material is placed in a magnetic field, the magnetic moments of the domains align along the direction of the applied magnetic field forming a large net magnetic moment. A residual magnetic moment exists even after the magnetic field is removed. In paramagnetic materials (such as gadolinium, magnesium, lithium, and tantalum) an atom has a net magnetic moment due to unpaired electrons but magnetic domains are absent. When the paramagnetic material is placed in a magnetic field, the magnetic moments of the atoms align along the direction of the applied magnetic field forming a weak net magnetic moment. These materials do not retain magnetic moment when the magnetic field is removed. In diamagnetic materials (such as copper, silver, gold, and most of the known elements) atoms have no unpaired electrons which results in zero net magnetic moment. These materials display a very weak response against the applied magnetic field due to realignment of the electron orbits when a magnetic field is applied. They do not retain magnetic moment when the magnetic field is removed. Antiferromagnetic materials (such as MnO, CoO, NiO, and CuCl_2_) are compounds of two different atoms that occupy different lattice positions. The two atoms have magnetic moments that are equal in magnitude and opposite in direction which results in zero net magnetic moment. Ferrimagnetic materials (such as magnetite Fe_3_O_4_ and maghemite γ-Fe_2_O_3_) are also compounds of different atoms residing on different lattice sites with antiparallel magnetic moments. However, in these materials, the magnetic moments do not cancel out since they have different magnitudes which results in a net spontaneous magnetic moment. When placed in a magnetic field, antiferromagnetic and ferrimagnetic materials show a behavior similar to that of ferromagnetic ones.

Magnetic nanoparticles (MNPs) are those nanoparticles (NPs) that show some response to an applied magnetic field. Nanotechnology allows physicists, chemists, material scientists and engineers to synthesize systems with nano sizes where the classic laws of physics are different at that small scale. As the size of the particle decreases, the ratio of the surface area to the volume of the particle increases. For nanoparticles, this ratio becomes significantly large causing a large portion of the atoms to reside on the surface compared to those in the core of the particle. For example, for a particle of 1 μm in diameter, nearly 0.15% of its atoms are on the surface, while for a particle of 6 nm in diameter nearly 20% of its atoms are on the surface.

As the size of the NPs decreases, the surface-to-volume ratio (and consequently the fraction of the surface atoms with respect to the bulk ones) increases. The large surface-to-volume ratio of the nanoparticles is the key factor to the novel physical, chemical, and mechanical properties compared to those of the corresponding bulk material. The physical properties include the optical, electric and magnetic properties. An example of chemical properties is the chemical reactivation rate. Examples of mechanical properties are strength and hardness. NPs of different types and sizes are now being synthesized via several physical and chemical methods and can be characterized and manipulated with several experimental techniques using atomic force microscopy, scanning tunneling microscopy and transition electron microscopy.

In this paper we discuss some of the main features of MNPs. This paper is not meant to cover all aspects of nanoparticles. We only shed light on selected papers within the large field of nanoparticles that we think are more relevant to their application in biomedicine and in particular to magnetic resonance imaging (MRI) as just an example out of many biomedical applications of MNPs.

It was shown that the magnetic moment per atom and the magnetic anisotropy of nanoparticles can be different than those of a bulk specimen [1]. Also, several other magnetic properties such as the Curie (*T_C_*) or Néel (*T_N_*) temperatures, and the coercivity field (*H_C_*) were found to be different than those for the bulk material [1]. It is well established that a bulk ferromagnetic material is composed of small regions, called magnetic domains. These magnetic domains resulted from a balance of several energy terms: the exchange energy, magnetocrystalline anisotropy, and the magnetostatic (or dipolar) energy [2,3]. The exchange energy tries to align all magnetic moments in the same direction, the magnetocrystalline anisotropy tries to orient magnetic moments along specific directions, and the magnetostatic energy tries to eliminate the magnetization in the material. In each domain the magnetic moments of atoms are aligned in one direction giving a net magnetization of each domain. The directions of magnetizations of the domains are different. Hence the net magnetization of a magnetic material resulted from the addition of the different magnetizations of all domains. It was found that magnetic domains in ferromagnetic crystals have a minimum (critical) size (around 100 nm) below which the ferromagnetic material cannot split up further into domains [4–6] and are called single domain particles. Thermal energy plays a major role in the magnetic instability of single domain magnetic particles [7]. The MNP might be composed of a single magnetic domain if its size decreases below a critical limit. It might also display a superparamagnetic [8,9] behavior as long as the temperature is above a particular temperature which is called the blocking temperature (*T_B_*). In the superparamagnetic state, the magnetic moments of the nanoparticles fluctuate around the easy axes of magnetization. Thus each one of the MNPs will possess a large magnetic moment that continuously changes orientation. When a magnetic field is applied, MNPs in the superparamagnetic state display a fast response to the changes of the magnetic field without remnant (residual) magnetization and without coercivity (the magnetic field required to bring the magnetization back to zero). Thus in the superparamagnetic state, a MNP behaves as a paramagnetic atom with a giant spin. At temperatures below the blocking temperature, the thermal agitation becomes small and will not be able to cause fluctuations in the orientations of the magnetic moments of the nanoparticles where they freeze in random orientations.

In order to analyze the properties of magnetic nanoparticles in a satisfactory manner, we need to obtain some geometric and magnetic data about them. Geometric data includes the size, shape, composition, and crystal structure of the nanoparticles. Magnetic data includes temperature dependence magnetization, saturation magnetization, remnant magnetization, coercivity, and blocking temperature.

The two main features that dominate the magnetic properties of nanoparticles and give them various special properties are:

(a) Finite-size effects (single-domain or multi-domain structures and quantum confinement of the electrons);
(b) Surface effects, which results from the symmetry breaking of the crystal structure at the surface of the particle, oxidation, dangling bonds, existence of surfactants, surface strain, or even different chemical and physical structures of internal “core” and surface “shell” parts of the nanoparticle.

In large magnetic particles, it is well known that there is a multi-domain structure where regions of uniform magnetization are separated by domain walls. The formation of the domain walls is a process driven by the balance between two factors:

(a) The external magnetostatic energy (*E_MS_*), which increases with the volume of the particle;
(b) The domain-wall energy (*E_dw_*), which increases with the interfacial area between domains.

If the particle size is reduced, there is a critical volume below which it costs more energy to create a domain wall than to support the external magnetostatic energy (stray field). Under this critical diameter which typically lies in the range of a few tens of nanometers (and depends on the type of material), the particle will consist of a single domain.

In a single-domain particle, all the spins are aligned in the same direction and the particle is uniformly magnetized. Because there are no domain walls to move, the magnetization will be reversed through spin rotation rather than through the motion of domain walls. This results in large coercivity of the nanoparticles.

There are two factors which result in high coercivity of small nanoparticles:

(a) Spin rotation instead of domain wall motion
(b) Shape anisotropy. Coercivity is smaller when the particles are spherical.

Shape anisotropy will also affect the estimation of the critical volume (below which the particle become single-domain). Spherical particles have small critical diameters compared with those of large shape anisotropy.

The spins in an isolated particle are held in a particular direction (not necessarily parallel to the applied field) via the magnetic anisotropy energy (which is caused by spin-orbital interactions of the electrons). If the particles are not isolated, other interactions will be involved. The anisotropy energy per particle is given by:

(1) $$E_{a}(\theta )=K_{eff}\;V\;sin^{2}\theta$$

(the leading term of the series expansion) where *V* is the volume of the particle, *K_eff_* the effective anisotropy constant, and *θ* is the angle between the magnetization and the easy magnetization axis of the particle. The maximum energy barrier is *K_eff_* V. This is the energy which separates the two energetically equivalent easy magnetization directions *i.e.*, the energy barrier to moment reversal (the size of this energy depends on many factors including magnetocrystalline and shape anisotropies). As the particle size, *V* decreases, *E_a_* also decreases. A point is reached when *E_a_* becomes small. If the temperature increases, the thermal energy, *E_th_* = *k_B_T* might exceed *E_a_*. This causes the particle magnetization to rotate freely resulting in the loss of magnetism in the absence of an applied magnetic field. The temperature at which this spin flipping occurs is called the blocking temperature, *T_B_*. The blocking temperature depends on the particle size and other factors. At *T* > *T_B_*, the isolated (non-interacting) single-domain particle becomes superparamagnetic. In this state, the magnetic moment of the particle behaves as that of a single atom (like a paramagnet) but with much larger magnitude (see Equation (2) below and the dependence of thermally activated flipping of magnetic moment on particle volume).

The relaxation time of the moment of a particle, *τ_N_* is given by the Néel expression where the factor *τ*_0_ ≈ 10^−12^ − 10^−9^*s* is weakly temperature dependent:

(2) $$\tau_{N}=\tau_{0}exp\;\left(\frac{K_{eff}\;V}{K_{B}T}\right)$$

If the time window of the measurement (*τ_m_*) is longer than the time needed for the particle’s magnetic moment to flip, the particle is said to be in a superparamagnetic state. On the other hand, if the experimental time scale is shorter than the moment flipping time, the particle is said to be in the blocked state. The blocking temperature (defined as the mid-point between these two states, where *τ_N_* = *τ_m_*) depends on several factors:

(a) The size of the particles
(b) The effective anisotropy constant, *K_eff_*
(c) The applied magnetic field
(d) The experimental measurement time

The blocking temperature can be estimated using d.c. magnetization measurements. These measurements involve the measurement of magnetization as a function of temperature in two different states. The first state is called the zero-field-cooled (ZFC) state, and the second one is called the field-cooled (FC) state. In the ZFC measurements, the sample is cooled from room temperature to a particular low temperature in the absence of magnetic field. Then a small magnetic field (about 100 Oe) is applied and the magnetization is measured as the temperature is being raised. As temperature increases, thermal energy will cause the moments to align along the direction of the applied magnetic field (*i.e*., overcoming anisotropy energy and freeing moments from being blocked at *T* < *T_B_*). The number of these aligned moments will increase as the temperature increases reaching a maximum at *T_B_*. As the temperature is raised above *T_B_*, the thermal energy becomes large enough (larger than that of the aligning field) to cause the magnetic moments to flip randomly which results in a suppression of the magnetization of the particle. The ZFC measurement will result in a peak in the magnetization *versus* temperature curve. This peak occurs at *T_B_*. If the sample being measured consists of particles of nearly equal sizes (small size distribution) then the particles of the sample will have nearly the same *T_B_* (small distribution of the blocking temperatures). Hence, the peak in the magnetization *versus* temperature curve will be sharp and *T_B_* for the particles is accurately estimated. If, on the other hand, the sample has a large size distribution, the particles of the sample will have a distribution of the blocking temperatures resulting in a broad peak magnetization *versus* temperature curve. In this case, the blocking temperature cannot be accurately estimated.

In FC measurements, the sample is cooled from room temperature to a particular low temperature in the existence of magnetic field. The magnetization is measured as the temperature is being cooled. At *T* > *T_B_*, thermal energy is large enough to randomize the magnetic moments in the particle leading to very small net magnetization. As the temperature is lowered, thermal energy will decrease and for some moments, it becomes smaller than that produced by the aligning field. This will cause some moments to align along the field direction leading to an increase in magnetization. As the temperature is decreases further, more and more moments will be frozen along the direction of the applied field. The magnetization of the sample is expected to keep increasing down to the lowest temperature of the experiment.

If the nanoparticles are not single-domain particles or/and if they are not isolated, then other interactions will be involved and the results of the ZFC and FC measurements will be complicated. Hence, the shape of the magnetization *versus* temperature plots in the ZFC and FC measurements can provide qualitative information about the size distribution and the strength of interaction among the particles making the sample.

As mentioned earlier, the magnetic anisotropy-supplied energy barrier *E_a_* must be overcome by the NP’s magnetic moment in order to change its orientation away from the easy axes of magnetization. The flipping of the NP magnetic moment vector (without changing the orientation of the particle itself) is the previously explained Néel relaxation (Equation (2)). In the absence of applied magnetic field this leads to the vanishing of the magnetization (e.g., *T* > *T_B_*). An applied magnetic field would supply the required energy to overcome *E_a_* which is then dissipated in the return to equilibrium. The second mechanism of electronic relaxation is the Brownian relaxation mechanism which involves the rotation of the particle itself against viscous forces. The time constant, *τ_B_*, characterizing this motion also increases with the particle volume, however, at a slower rate than the Néel relaxation time constant according to the following equation

(3) $$\tau_{B}=\frac{3\;V\;\eta }{K_{B}\;T}$$

where *η* is the viscosity of the liquid containing the particles. The total magnetic relaxation *τ_eff_* time is then given by

(4) $$\frac{1}{\tau_{eff}}=\frac{1}{\tau_{N}}+\frac{1}{\tau_{B}}$$

It can be seen that if *τ_B_* < *τ_N_* (as for large NP with radius > 15 nm) then it is the viscous component which dominates magnetic relaxation. Both of these mechanisms have direct effects on two biomedical applications (MRI and Magnetic Hyperthermia) of magnetic NP. The former will be discussed in more details in this article.

## 2. Surface Effects

Because of the small size of nanoparticles, large fractions of all the atoms in a nanoparticle are surface atoms [10]. Since the ratio of surface atoms to the bulk atoms is large, surface contribution to magnetization becomes significant. Generally, a magnetic nanoparticle is considered to consist of a single domain particle with uniaxial anisotropy. The orientation of its magnetic moment points either “up” or “down” in a zero field along the easy axis. However, the surface atoms experience different environments than those in the core of the particle. There are several types of defects that exist on the surface such as atomic vacancies, changes in the atomic coordination, dangling bonds and lattice disorder. These surface defects result in uncompensated disordered spins at the surface of the nanoparticle leading to surface magnetization (ferromagnetism or antiferromagnetism). The surface magnetization is contributed by the surface uncompensated spins, which depend on the size of the particle and on the degree of surface disorder [11,12]. Because of surface effects, ferromagnetism could be a universal feature of nanoparticles and their oxides. For example, nanoparticles of nonmagnetic materials such as cerium oxide and aluminum oxide were found to display magnetic hysteresis loops at room temperature. Nanoparticles of metal nitrides, such as niobium nitride were found to exhibit ferromagnetism effects. Nanoparticles of some superconductors in the normal state were found to show ferromagnetism. The smaller the nanoparticle, the larger is the ferromagnetism effect. High field hysteresis and relaxation of the magnetization could result due to irreversible reorientations of the surface spins [13]. Using molecular dynamic modeling, [14] Nunes *et al*. considered the structural relaxation of spinel ferrite nanoparticles. They predicted non-uniform strains in the surface layers, with an average expansion of a few percent compared to bulk. They suggested that such an expansion might result in a stress-induced anisotropy field of up to 70 kOe, which could account for some of the anomalous magnetic behavior of ferrite nanoparticles. Kodama *et al*. proposed that the canted spins in ferrite nanoparticles freeze into a spin glass-like phase at temperatures below 50 K [15–17]. Thus, the surface spins have multiple configurations for any orientation of the core magnetization. This model accounts for the reported surprising decrease of the magnetization of the nanoparticle as the size of the nanoparticle increases [18,19] as well as the remarkable irreversibility and time dependent moment in high fields [15,20]. Several magnetic effects could also result from the finite size effect of nanoparticles. These could include:

(a) The existence of randomly oriented uncompensated surface spins.
(b) The existence of canted spins.
(c) The existence of a spin-glass-like behavior of the surface spins.
(d) The existence of a magnetically dead layer at the surface.
(e) The enhancement of the magnetic anisotropy which results from surface anisotropy.

It should be emphasized that surface effects can lead to a decrease or an increase in the magnetization of nanoparticles. It was reported that the magnetization of oxide nanoparticles decreases for some oxide nanoparticles [13]. On the other hand, the magnetization of some metallic nanoparticles (cobalt) was reported to increase [21]. The reduction of magnetization of oxide nanoparticles was attributed to the existence of a magnetically dead layer on the particle’s surface, the existence of canted spins, or the existence of a spin-glass-like behavior of the surface spins [13]. Several experimental studies reported an increase in the effective magnetic anisotropy due to surface effects [22–25]. Computational studies also reported different anisotropy and magnetic moment at the surface of magnetic clusters embedded in matrices [26]. Synchrotron radiation studies revealed that both spin and orbital moments at the surface are different from those of the bulk counterparts [27]. Thermal measurements also reported that the structure of nanoparticles and the strength of their surface anisotropy control their magnetic properties [28].

The total magnetization of the nanoparticle is suggested to be composed of two components; a component due to the surface spins and a component due to the core of the particle. Thus the magnetization of the magnetic nanoparticles can be modeled via a core-shell (or core-surface) magnetic model leading to another type of magnetic interaction at the interface between the core and the shell. In nanoparticles of antiferromagnetic (AFM) or ferrimagnetic (FIM) materials, this interaction occurs at the interface between the ferromagnetic (FM) surface and the AFM (or FIM) core. In some FM nanoparticles, the surface of the metal usually oxidizes in air and forms an AFM metal-oxide shell around the FM metal core. Thus, there will be an interaction between the FM core and the AFM shell. The core-shell interaction is called the exchange bias or exchange coupling. The exchange coupling provides an additional magnetic anisotropy to help align the ferromagnetic spins in certain directions. The exchange coupling is known to vanish above a critical temperature called the blocking temperature, but no satisfactory understanding of this interaction at the microscopic level exists. Because the AFM (or FIM) core of a nanoparticle has a small net bulk magnetization, it serves to pin (or stabilize) the magnetization of the ferromagnetic surface without adding additional magnetization to the system. At the interface, the spins of the AFM core exert a microscopic torque on the spins of the ferromagnetic surface, to pin them in their original position. Thus, the magnetic field (or temperature) needed to completely reverse a ferromagnetic layer will be larger if it is in contact with an AFM core, because an extra energy is needed to overcome the microscopic torque.

In order to obtain a satisfactory understanding of the theory of exchange bias, it is essential to understand the atomic interface structure [29]. The interfacial exchange coupling in Mn_3_O_4_-MnO and Mn_3_O_4_-Mn FIM-AFM core-shell nanoparticles was reported to depend on the atomic structure and strain at the interface [30,31]. The authors reported an inversion of the order temperature of the Mn_3_O_4_-MnO core-shell system, where the Curie temperature, *T_C_* of the FIM Mn_3_O_4_ core is smaller than the Néel temperature, *T_N_* of the AFM MnO shell. The authors reported that in Mn_3_O_4_-Mn core-shell nanoparticles, the two phases of Mn and Mn_3_O_4_ are in close contact with low interface defect concentration and low strain. The result was a large interfacial exchange coupling leading to a large exchange bias field [30]. On the other hand, a larger interfacial defect concentration and high strain occurred at Mn_3_O_4_-MnO core-shell interface. The result was a smaller interfacial exchange coupling leading to a smaller exchange bias field [31].

In a very interesting study, Berkowitz *et al*. investigated the magnetic and microstructural properties of core-shell nanoparticles which consist of cores of antiferromagnetic MnO and shells of ferromagnetic Mn_3_O_4_[32]. This arrangement is opposite to the usual FM-AFM core-shell arrangement. In addition, the magnetic order temperatures were also found to be inverted where *T_N_* for the AFM core is larger than the *T_C_* for the FM shell. The authors called this new arrangement a “doubly inverted core-shell system” [32]. It was found that the MnO-Mn_3_O_4_ interface is ordered, which implies strong interfacial coupling. Hence, large exchange field (*H_E_*) values were obtained at temperatures below *T_C_* of the FM Mn_3_O_4_ shell.

In a recent and interesting study [33], the dimensions of the core and shell of monodispersed FeO-Fe_3_O_4_ AFM-FIM core-shell nanoparticles of the same total diameter (of 35 nm) were tuned via controlling the oxidation temperature and time. It was found that the coercivity, *H_C_* and the exchange field, *H_E_* increase when the dimension of the shell decreases and that of the core increases. Since all the nanoparticles used in the study have the same total diameter, this indicates that it is the core-shell interface area which determines *H_C_* and *H_E_*. This behavior could be understood to be the result of two competing factors. The first factor is the exchange coupling (or exchange anisotropy) at the core-shell interface which is expected to increase when the effective interface area increases. The second factor is the magnetostatic effect (or magnetostatic anisotropy) which competes with interface exchange coupling. As the shell becomes thinner and the core becomes larger, the effective core-shell interface becomes larger and the exchange coupling increases resulting in large *H_C_* and large *H_E_*. On the other hand, core magnetostatic anisotropy increases since it scales with the core volume. Hence, the efficiency of the shell magnetic moments to pin those of the core decreases, resulting in small *H_C_* and *H_E_*. Based on the results in [33], the core-shell interface exchange coupling outweighs the role of magnetostatic anisotropy giving a net increase in *H_C_* and *H_E_*. A small core and large shell dimensions lead to opposite behavior, with smaller *H_C_* and *H_E_*. The largest competition between the magnetostatic anisotropy and the exchange coupling occurs when the shell is the thinnest and the core is the largest. In this situation, the authors [33] suggested that domain wall nucleation occurs and magnetization reversal occurs via domain wall propagation leading to the observed nonsymmetrical hysteresis loop. On the other hand, the smallest competition between the magnetostatic anisotropy and the exchange coupling occurs when the shell is the thickest and the core is the smallest. In this case, magnetization reversal occurs mainly via magnetization rotation leading to a symmetric hysteresis loop.

A vertical shift in the *M*–*H* curve such that both the descending and ascending remnant magnetizations are positive was also reported [33] and was attributed to the presence of uncompensated spins at the core-shell interface. When cooling in a zero applied field, these spins are aligned antiferromagnetically with the AFM core and contribute no net magnetization. However, when the particles are cooled from above the Néel temperature) under an applied field, these spins are aligned with the field but still pinned by the AFM core, thus maintaining a preferred direction of magnetization. The authors [33] also attributed the lack of saturation at low temperatures mainly to be due to the three competing factors which the uncompensated spins experience at the core-shell interface. These are the magnetostatic effect, the exchange coupling with the core moments, and the exchange coupling with shell moments.

However, according to the findings in [30,31], the existence of defects at the core-shell interface (which results in the existence of interfacial uncompensated spins) will result in a weak contact at the interface and leads to a small interfacial exchange coupling.

The coercivity of the FePt-Fe_3_O_4_ ferromagnetic-ferrimagnetic (FM-FIM) core-shell nanoparticles was found to depend on the volume ratio of the core and shell phases, not on the actual size or thickness of the core and the shell [34]. The intimate contact between the FePt core and Fe_3_O_4_ shell was reported to lead to an effective interface exchange coupling, which results in cooperative magnetization switching of the two phases. They reported that magnetic properties of these core-shell nanoparticles can be tailored by controlling the core-shell dimensions, and by tuning the material parameters of both core and shell. The situation of FePt-CoFe_2_O_4_ FM-FIM core-shell nanoparticles was found to be different with larger *H_C_*. The authors attributed the increase in *H_C_* to the larger magnetocrystalline anisotropy of the CoFe_2_O_4_ phase compared with that of the Fe_3_O_4_ phase [34].

## 3. Ferrite Nanoparticles

Ferrites were discovered thousands of years ago. The first compass was made of magnetite (Fe_3_O_4_). Magnetic properties are interesting from the fundamental and technological points of view. Small enhancements in some magnetic properties such as permeability, coercivity, or saturation magnetization can have great impact on economy.

The unit cell of ferrite spinel structure (with lattice parameter value, *d* ~0.84 nm) is formed by 32 O^2−^ anions and 24 cations (Fe^2+^, Zn^2+^, Co^2+^, Mn^2+^, Ni^2+^, Mg^2+^, Fe^3+^, Gd^3+^). There are 96 possible positions for cations in the unit cell (64 tetrahedral and 32 octahedral positions). The octahedral sites are larger than the tetrahedral sites. Only 8 tetrahedral positions and 16 octahedral positions are occupied by cations (divalent or trivalent).

The spinel structure generally has the form (A)[B_2_]O_4_ which is described as a cubic closed-pack of oxygen ions. In this structure, the round brackets represent the tetrahedral interstitial (A) sites and the square brackets represent the larger octahedral interstitial [B] sites. Both the tetrahedral and octahedral interstitial lattice sites are occupied by cations. When all the divalent cations occupy the tetrahedral sites while all the trivalent cations occupy the octahedral sites, the structure is called the normal spinel structure of the form (A)[B_2_]O_4_. When all the divalent cations occupy the octahedral sites while half of the trivalent cations occupy the tetrahedral sites and the other half occupy the octahedral sites, the structure is called the inverse spinel structure of the form (B)[AB]O_4_.

**Figure 1:**
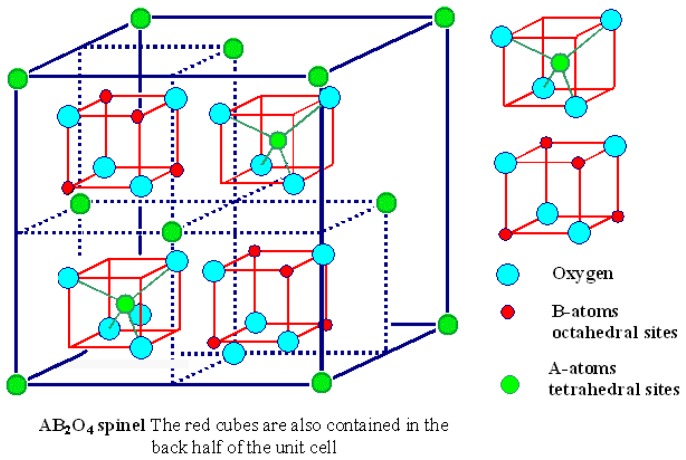
The spinel structure of ferrites is shown indicating the tetrahedral and octahedral sites. This figure is copied from the following website http://www.tf.uni-kiel.de/matwis/amat/def_en/kap_2/basics/b2_1_6.html.

The general structure of the ferrites spinel structure can be written as (Me^2+^)[Fe^3+^_2_]O_4_. Here Me^2+^ represent metals ions such Fe^2+^, Zn^2+^, Co^2+^, Mn^2+^, Ni^2+^, Mg^2+^. The Fe^3+^ cations serve as the trivalent cations while Me^2+^ cations serve as the divalent cations. In the spinel structure of ferrites (Figure 1), the Fe^3+^ atoms occupy the octahedral positions (small red spheres) while Me^2+^ occupy tetrahedral positions (green spheres). In some situations, the ferrite structure might have a structure that is in between the normal and inverse spinel structures. Hence, in general, the formula of the ferrite spinel can be written as (Me^2+^_1−_*_x_*Fe^3+^*_x_*)[Me^2+^*_x_*Fe^3+^_2−_*_x_*]O_4_. The variable *x* is called the degree of inversion and represents the proportion of Fe^3+^ occupying the tetrahedral sites. When *x* = 0, we obtain (Me^2+^)[Fe^3+^_2_]O_4_, which is the normal spinel structure. When *x* = 1, we obtain (Fe^3+^)[Me^2+^Fe^3+^]O_4_, which is the inverse spinel structure. It is known that in the bulk form of the ferrite materials, the divalent cations Fe^2+^, Co^2+^, Mn^2+^, Ni^2+^, Mg^2+^ prefer to occupy the larger octahedral lattice sites while the Zn^2+^ prefers to occupy the smaller tetrahedral lattice sites [35,36].

In the bulk form, ZnFe_2_O_4_ has a normal spinel structure where nearly all Zn^2+^ cations are incorporated at the tetrahedral lattice sites and Fe^3+^ cations are at the octahedral sites, giving the form (Zn^2+^)[Fe^3+^_2_]O_4_. However it was reported that the size and synthesis method of ferrite nanoparticles can result in structure, composition, cation distribution and magnetic properties that are not observed in their bulk forms [36–44].

In spinel ferrites, all the cations at the octahedral lattice sites have magnetic moments which are oriented in the same direction. At the same time, all the cations on the tetrahedral lattice sites have magnetic moments which are oriented in the same direction but antiparallel to that of the cations at the octahedral lattice sites [45]. The net magnetization of the spinel ferrites is due to the difference in the magnetic moments of the cations at the octahedral lattice sites and those at the tetrahedral lattice sites. The source of the magnetic moment of cations (of the transition metals) is the spin magnetic moment of the unpaired 3*d* electrons.

Néel postulated that magnetic moments of spinel ferrites are the sum of magnetic moments of individual sublattices. These are: sublattice A consisting of cations in tetrahedral positions and sublattice B with cations in octahedral positions. Exchange interaction between electrons of cations in these sublattices has different value. Usually interaction between magnetic cations on sublattice A and those on sublattice B (AB-interaction) is the strongest. The AB exchange interaction occurs across the oxygen ions separating the cations at sublattice A from those at sublattice B. Hence, this interaction is called the super-exchange interaction (A-O-B). The AA-interaction is much weaker than the AB-interaction while the BB-interaction is the weakest [45].

The magnetization of zinc ferrite nanoparticles was found to be much higher than its value in the bulk form [46]. In the bulk form, ZnFe_2_O_4_ shows paramagnetic behavior due to its normal spinel structure, with Zn^2+^ ions incorporated almost exclusively at tetrahedral sites. When ZnFe_2_O_4_ is prepared in the form of nanoparticles, it becomes ferrimagnetic due to a partial migration of Zn^2+^ ions to the octahedral sites [46–51]. This migration results in a distribution of the magnetic Fe^3+^ cations and the non-magnetic Zn^2+^ cations among the octahedral and tetrahedral lattice sites which results in changes in their magnetic properties. The magnetization of zinc ferrite nanoparticles were found to decrease as the Zn/Fe ratio becomes larger than 0.5 [52]. If we assume that the extra non-magnetic Zn^2+^ cations occupy the tetrahedral lattice sites, this will decrease the net magnetic moment of the tetrahedral sublattice. The difference between the net magnetic moment of the two sublattices becomes larger leading to an increase in the net magnetization of the nanoparticle, which is opposite to what was observed. Hence, the decrease of magnetization of the zinc ferrite nanoparticles as the Zn/Fe ratio becomes larger than 0.5 occurs because the extra non-magnetic Zn^2+^ cations occupy the octahedral lattice sites. This results in the decrease of the net magnetic moment of the octahedral sublattice. The difference between the net magnetic moment of the octahedral and tetrahedral sublattices becomes smaller resulting in the observed decrease in the net magnetization of the nanoparticle. Point defects might also lead to further decrease in magnetization of the zinc ferrite nanoparticles. When Zn/Fe ratio becomes larger than 0.5, large number of point defects such as oxygen vacancies will appear in the spinel structure. Oxygen vacancies will disturb the A-O-B super-exchange interaction between the magnetic cations in the two sublattices, which results in the decrease of the net magnetization of the nanoparticle.

Despite several studies on the magnetic properties of these ferrite nanoparticles, their magnetic behavior is not yet well understood. Recent studies on ferrite nanoparticles [15,16,20,53] and Fe nanoparticles coated with magnetic oxides [45,52,54] show a complex picture of the magnetic state of the nanoparticles. These studies suggest the occurrence of surface structure defects, which could lead to magnetic disorder extending into the core within a layer of a given thickness. Thus the most accepted theoretical model to explain some magnetic properties such as the decrease of magnetization of nanoparticles (compared with that of the bulk material) is based on a bulk-like ferromagnetic core and a shell composed of disordered moments [55,56]. Several studies show inconsistency of the results of the shell thickness of nanoparticles and no correlation between particle size and shell thickness was obtained [46–48]. Similar inconsistent magnetic behaviors of the nanoparticles were also reported [49–51]. These variations of results could be because the magnetic behavior of magnetic nanoparticles is influenced by several factors such as size distribution, surface and internal defects, and inter-particle dipolar and exchange interactions. The exchange coupling between the surface and core gives rise to a variety of spin distributions within the nanoparticle. Kodama *et al*. [15,16] proposed that the canted spins freeze into a spin glass-like phase at temperatures below 50 K. The particle size, magnetic properties, and surface properties of the nanoparticles are reported to be influenced by the method of synthesis employed [57].

It is important to mention that almost half a decade ago, a small number of Fe^3+^ were suggested to occupy the tetrahedral A sites [58,59] and a small degree of inversion (*x* = 0.04) was reported in bulk ZnFe_2_O_4_[60,61]. When ZnFe_2_O_4_ ferrite was prepared as nanoparticles, a significant proportion of Zn^2+^ ions were found to enter the zinc-ferrite structure at the octahedral sites, resulting in a non-stoichiometric ferrite [54]. The lattice parameters of the stoichiometric ZnFe_2_O_4_ nanoparticles were measured to be larger than those for the bulk material [52] suggesting that the nanoparticles have different crystal structure than that of the bulk material. This difference between lattice structure of the bulk and nanoparticles of ZnFe_2_O_4_ material suggested that the cations in the zinc ferrite nanoparticles might have a distribution, over the octahedral and tetrahedral lattice sites, which is different than that in the bulk material. The larger lattice parameter of the nanoparticles was suggested to be due the incorporation of the octahedral lattice sites by some Zn^2+^ ions which have larger size than the Fe_3+_ ions. The small size and large surface to volume ratio of nanoparticles is believed to be result in this flexibility of the composition of the ZnFe_2_O_4_.

The possibility of incorporating Zn^2+^ ions at the octahedral sites of the spinel structure of nanoparticles provides an opportunity to increase the number of Zn^2+^ ions in the spinel structure compared to that of the stoichiometric ZnFe_2_O_4_ bulk composition. Zinc ferrite nanoparticles were found to allow large deviations from the familiar stoichiometric ZnFe_2_O_4_ composition of the bulk form with Zn/Fe = 0.5. Particles with a ratio of Zn/Fe that is larger than 0.5 were synthesized [52]. The possible variations of the Zn/Fe ratio above the stoichiometric value of 0.5 were reported to have some effects on the interior structure of these ferrites. The lattice parameter of the spinel structure of the ZnFe_2_O_4_ nanoparticles was found to increase with increasing Zn/Fe ratio up to the value of Zn/Fe = 0.8. Above the ratio of Zn/Fe = 0.8, ZnO appeared as a secondary phase [52]. This suggests that smaller ZnFe_2_O_4_ nanoparticles can allow significant deviations from the stoichiometric composition while maintaining the single phase spinel structure. The larger lattice parameter with the increasing Zn/Fe ratio is mainly due to the larger size of the Zn^2+^ ions compared to the Fe^3+^ ions. The non-stoichiometry of the ferrite nanoparticles can be structurally compensated by the formation of point defects in their structure such as oxygen vacancies and cation vacancies at the octahedral lattice sites [52]. Hence the non-stoichiometry of the composition of the zinc ferrites results in several factors such as changes in lattice parameter, the appearance of secondary phases, and the appearance of point defects.

The possibility of incorporating Zn^2+^ ions at the octahedral sites (of inversion for ZnFe_2_O_4_ nanoparticles) results in non-stoichiometry of the composition of zinc ferrite nanoparticles [52]. But the degree of inversion was reported to depend on the size of the particles, synthesis methods and conditions [46,50,56,57]. The synthesis temperature was also found to have a strong influence on the size and lattice parameters of the Zn-ferrite [52] as well as on the crystallinity of Mn-Zn ferrite nanoparticles [62].

The Co-ferrite has the stoichiometry of CoFe_2_O_4_ in the bulk form. In the nanoparticle form, it was shown that the distribution of the cations within the spinel lattice of the nanoparticles is changed [55,56,63–65].

In Mn_0.5_Zn_0.5_Fe_2_O_4_ spinel ferrite nanoparticles, it was reported that a migration of Mn^2+^ and Zn^2+^ ions from the tetrahedral lattice sites to the octahedral sites of the spinel lattice was compensated by the corresponding migration of the Fe^3+^ ions [51]. The average Mn valence in the nanoparticles was found to be higher than 3^+^[51]. High resolution electron microscopy (HREM) images showed that the edge of agglomerate of nanoparticles with size of 8 nm display regular crystallinity. However, the edge of agglomerate of nanoparticles with size of 1.5 nm appeared to be amorphous with small islands of periodicity for particular nanoparticles [51]. This structure significantly influenced the magnetic properties of these nanoparticles. The *H-M* measurements revealed that nanoparticles with sizes larger than 3 nm display ferrimagnetism. The saturation magnetization was not reached at room temperatures at high magnetic fields for all nanoparticles. This observation was attributed to a magnetically inactive surface layer which becomes more prominent with decreasing size [66]. The magnetization values measured at 10 kOe were found to decrease as the size of the NPs decreases. Nanoparticles of sizes less than 3 nm were found to become paramagnetic indicating that the inactive surface layer becomes dominant [51].

In a study on nanostructure nickel ferrite (NiFe_2_O_4_) of grain sizes of 13, 20, 26, and 51 nm, the smallest and largest grain-sized samples revealed surface spin canting and change in coordination of the iron ions at tetrahedral and octahedral sites with reduction in grain size [67]. A lower value of magnetization for the samples of lowest grain size was also obtained and was attributed to a structural transformation of the sample from inverse to mixed spinel [67].

Hence, the size of ferrite nanoparticle, the synthesis methods, and conditions can lead to different degrees of inversion in the spinel structure resulting in non-stoichiometry of the composition of these nanoparticles. This results in changes in lattice parameter, the appearance of secondary phases, and the appearance of point defects. This results in changes in the magnetic properties of the ferrite nanoparticles. It is, therefore, very important to realize that magnetic properties of the ferrite nanoparticles can be tuned by controlling the synthesis methods and size of particles.

Finite size and surface effects on the magnetic properties were investigated in Mn_0.5_Zn_0.5_Gd*_x_*Fe_(2−_*_x_*_)_O_4_ ferrite nanoparticles, with *x* = 0.02, 0.05, 0.11, 0.15, and 0.2 [68]. The particles’ sizes ranged from 4 to 10 nm. Nonmonotonic behavior of Curie temperature, *H_C_*, the spontaneous magnetization, *σ*, the coercivity field, *H_C_*, the irreversibility field, *H_irr_*, and remnant magnetization, *M_R_*, with the size of particles were reported and discussed. Although finite size effects might have significant role on the magnetization of nanoparticles, considering finite size effects alone cannot account for the nonmonotonic behavior of the magnetic properties *versus* the size of the particles, “*d*”. The authors proposed two models that could account for some of the nonmonotonic magnetic behavior in these ferrite particles [68]. The first model considers possible variations in the structural compositions of non-interacting particles with no surface spin effects. The structure of the Mn_0.5_Zn_0.5_Gd*_x_*Fe_(2−_*_x_*_)_O_4_ ferrite is known to be normal spinel [35,69,70]. Because of the Gd^3+^ large ionic radii, addition of Gd^3+^ cations results in their occupancy of the octahedral sites [71–74]. Because the Gd^3+^ cations possess large magnetic moment, the initial addition of these ions is expected to enhance the net magnetic moment of the octahedral atoms and accordingly the total magnetization will increase. Further addition of Gd^3+^ cations will lead to a decrease in the distance between these ions and the oxygen anions, which strengthens the interaction between cations at the octahedral sites. Hence, the cations at the octahedral sites will no longer have their moments parallel to each other where some of them will have their moments aligned anti-parallel leading to a reduction in the net magnetic moment of the octahedral cations and to a suppression of the total magnetization. However, the subsequent rise in the magnetization with more addition of Gd^3+^ is not simple to explain using the variations in the structural compositions.

In the second model, the authors proposed that effects on the particle surface (shell) and the exchange coupling at the core-shell (surface) interface could help to explain the nonmonotonic magnetic behavior *versus* “*d*” [68]. The total magnetization of a nanoparticle is suggested to be composed of two parts; the magnetization due to the unpaired surface spins and the magnetization due to the core of the particle. The net magnetic moment of the particle is determined by considering several factors such as the magnetic interaction among the surface spins, the exchange coupling at the core-shell interface, the magnetic anisotropy of the cores and the surface anisotropy. The surface anisotropy could strongly influence the magnetization behavior of nanoparticles. The strong surface anisotropy and the shape of nanoparticles were found to result in a horizontal shift in the exchange bias effect [75]. Core-shell exchange interaction and surface anisotropy were found to play significant roles in determining some magnetic properties of Fe(Co)NiB ferromagnetic nanoparticles [76]. The structural modifications at the boundaries of the ferrimagnetic nanoparticles, such as vacancies, broken bonds, may induce enough frustration, which leads to different canted magnetic structures [43]. The canted surface spins may freeze giving rise to a glassy state at low temperatures. One of the important features characterizing the surface spin-glass state in nanoparticles is the flattening of the FC magnetization at low temperatures [77] which is observed in some of the samples [68]. These authors suggested that the nonmonotonic magnetic behavior observed in the samples could be attributed to the disordered surface spins that freeze at low temperatures in a disordered state leading to spin glass-like behavior. The substitution of Fe^3+^ cations by the larger Gd^3+^ cations in the Mn_0.5_Zn_0.5_Gd*_x_*Fe_(2−_*_x_*_)_O_4_ is suggested to enhance the production of surface defects and frustration which leads to the formation of surface spins and spin canting.

The main origin of spin glass-like behavior in ferrite nanoparticles could be due to strong inter-particle interactions or due to surface spin effects within individual particles. Some researchers suggested that the main contribution to the existence of this glassy behavior is the strong magnetic inter-particle interactions [78–80].

## 4. Applications of Magnetic Nanoparticles

MNPs are of great interest for a wide range of disciplines, such as magnetic fluids [81], catalysis [82], biomedicine [75,83–85], magnetic energy storage [86], information storage and spintronics [87]. They are used to enhance the capacity of magnetic storage devices such as magnetic tapes, and computer hard discs [88]. Magnetic nanoparticles can also be used as giant magneto-resistance (GMR) sensors. In the medical field MNPs are used as contrast agents (CA) to enhance the contrast in MRI [89–92]; in tumor therapy where they can be selectively introduced into the tumor cells and then their temperature is increased using an oscillating magnetic field to reach near 43 °C (this temperature is known to make the tumor cells more sensitive to radiation and other treatment modalities) [93]; and finally used as site-specific drug delivery agents which involves immobilizing the drug on magnetic materials under the action of external magnetic field. Ferrofluids of nanoparticles were used in the treatment of solid tumours [94] and later used as magnetoliposomes in drug and/or peptide delivery systems and in the diagnosis and treatment of diseases [95–97]. Magnetic nanoparticles can also be used in water treatment [98]. There is still a huge potential for magnetic nanoparticles to be implemented in a wider range of applications, which requires advances in the synthesis methods in order to produce nanoparticles with specific sizes, very narrow size distribution and well controlled magnetic properties. The application of magnetic nanoparticles also highly depends on the stability of the particles. For example, magnetic moments of nanoparticles become unstable with temperature when the size of the nanoparticles becomes very small. Hence, stabilization of the magnetic moments is necessary to enhance the storage capacity of magnetic storage devices.

Oxidization of the surface of the nanoparticles could also be a major issue for long time exposure to air where the nanoparticle may be fully oxidized. Thus protection of the magnetic nanoparticles against oxidization and corrosion should be resolved.

Some of these challenges can be resolved by controlling the size, composition, and structure of the magnetic particle or by modifying the surface of the particle by coating with starch, dextran or polyethylene glycol (PEG) [99,100] as will be discussed later on in this article.

### 4.1. Properties for Medical Applications

The implementation of MNPs in the fields of biomedicine is varied [75,83–85]. The synthesis of magnetic nanoparticles for these applications is not simple, since it necessitates the control of the particle size, shape, stoichiometry and the surface structure. Several methods such as co-precipitation, thermal decomposition and reduction, micelle synthesis, and hydrothermal synthesis have been used to produce small nanoparticles with nearly uniform size distribution but with less control of the surface structure.

As the size of nanoparticles is reduced, deviations from bulk magnetic properties appear. The new properties are attributed to surface magnetization effects and to finite-size effects. For biomedical applications, several properties of nanoparticles must be attained [101]:

(a) The magnetic nanoparticles should be biocompatible and non-toxic.
(b) The magnetic nanoparticles are preferred to be sufficiently small (10–50 nm). This will have several advantages:
  (i) The nanoparticles will preserve their colloidal stability and resist aggregation if their magnetic interaction is reduced. This can be achieved if their magnetism disappears after removal of applied magnetic field. This superparamagnetic behavior is only achievable under certain particle size and above the blocking temperature.
  (ii) The dipole-dipole interactions scale as *r*^6^ (*r* is the radius of the particle). Hence, the dipolar interactions become very small when the particle size becomes very small. This will serve to minimize particle aggregation when the field is applied.
  (iii) Decreasing size means larger surface area for certain volume (or mass) of the particle. The efficiency of coating (and also the attachment of ligands) will improve leading to even more resistance to agglomeration, avoidance of biological clearance and better targeting.
  (iv) Being very small, the particles can remain in the circulation after injection and pass through the capillary systems of organs and tissues avoiding vessel embolism.
  (v) The magnetic particles will be stable in water at pH = 7 and in a physiological environment.
  (vi) Precipitation due to gravitation forces can be avoided with small particles.
(c) The magnetic particles must have a high saturation magnetization. This is an important requirement for two reasons:
  (i) The movement of the particles in the blood can be controlled with a moderate external magnetic field.
  (ii) The particles can be moved close to the targeted pathologic tissue.

### 4.2. Biocompatibility and Toxicity of the Magnetic Nanoparticles

*In vivo* applications require MNP to be biocompatible, stable, nontoxic, and monodispersed which requires controlling particle material, size and coating properties [90,102–104].

The magnetic nanoparticles which are used in biomedical applications are mainly iron oxide particles such as magnetite (Fe_3_O_4_) and its two oxidized products, tetragonal maghemite (γ-Fe_2_O_3_) and hexagonal hematite (α-Fe_2_O_3_). These natural materials are found in many biological systems [105–108]. Metallic magnetic materials such as iron, cobalt and nickel are toxic, and susceptible to oxidation. Hence, it is important to chemically stabilize the uncoated magnetic nanoparticles against degradation which occurs due to oxidation (corrosion) and acid erosion.

For NPs to be able to approach and interact with the biological molecules they have to be able to evade the reticuloendothelial system (RES). Immediately after being injected into the blood stream the NPs are coated with blood plasma proteins. This process, known as opsonization, renders the NPs susceptible for identification and later removal by phagocytic cells. Hydrophilic NPs (due to their coating chains such as derivatives of dextran and PEG) can resist opsonization and therefore increase their circulation time and enhance the probability of reaching their target cells [109,110]. Furthermore, these polymers can improve colloidal stability by steric effects. For example, the clearance rate of dextran-coated liposomes was found to be dependent on the density of the dextran molecules on the liposome surface [111]. The extended configuration on the NP surface improves the ability of the NPs to evade the macrophage system and therefore enhances permeability and retention processes [112,113].

Some protection methods include:

(a) Coating the NPs with organic species (including surfactants or polymers).
(b) Coating the NPs with an inorganic layer (such as silica or carbon).

Despite the wide use of superparamagnetic iron oxide (SPIO) nanoparticles in biomedical applications, questions remain regarding the effect of nanoparticle size and coating on nanoparticle cytotoxicity. Yu *et al*. [114] studied NP uptake and cytotoxicity by exposing porcine aortic endothelial cells to 5 and 30 nm diameter iron oxide NP coated with either the polysaccharide dextran, or the polymer polyethylene glycol (PEG). Uncoated NP of both sizes induced a more than 6 fold increase in cell death at the highest concentration (0.5 mg/mL) and led to significant cell elongation, whereas cell viability and morphology remained constant with coated nanoparticles. Significant reactive oxygen species (ROS) formation was induced with only the uncoated large (30 nm) NP. Furthermore, NPs were more toxic at lower concentrations when cells were cultured within 3D gels. Cytotoxicity, it seems, was reduced by using coating, however, different mechanisms may be important for different size NP.

### 4.3. Agglomeration and Particle Coating

In biomedical applications the NP suspension may be delivered to the site of application intravenously or by direct localized injection. Both of these methods require that the particles do not agglomerate and therefore do not inhibit their own distribution. This desired stability can be aided by reducing the size of the NP or by modifying their surface chemistry.

Modifying the surface chemistry of the particles by coating them with high molecular weight polymers [69,115] has the most direct effect of increasing the hydrodynamic size of the particles (therefore reducing its diffusional properties) and modifying transport and biodistribution properties. Coating also plays a role in the colloidal stability of the particles against aggregation and gravitational settling by reducing the inter-particle dipole-dipole forces [116–121]. The most common coatings are derivatives of dextran [122,123], polyvinylalcohol (PVA), polyethylene glycol [PEG], *etc.* [124]. For example, it was shown [125] that simply reducing the terminal sugar in a dextran coating has a significant effect not only on coating stability but also on particle size and magnetic properties. Micelle-based phospholipid-PEG coating was employed to encapsulate monocrystalline iron oxide nanoparticles (MIONs) [126]. Their advantage over the study [125] was that they were able to control thickness of the coating layer independently of the magnetic core. This was achieved simply by varying the length of the PEG polymer that is in the outer most surface of the particle. Eudragit and chitosan were also used by others as coating materials and drug releasing regulator [127].

**Figure 2:**
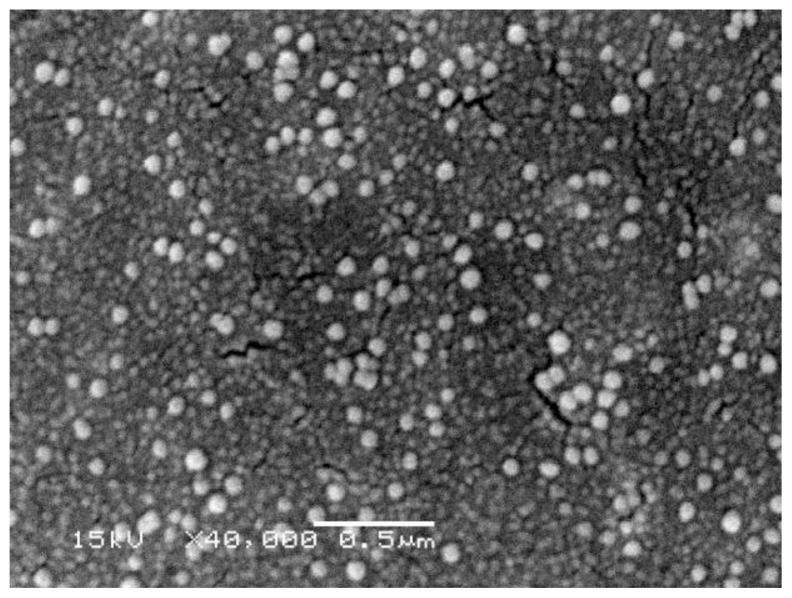
SEM of well dispersed spherical nanoparticles suspended on agarose gel.

**Figure 3:**
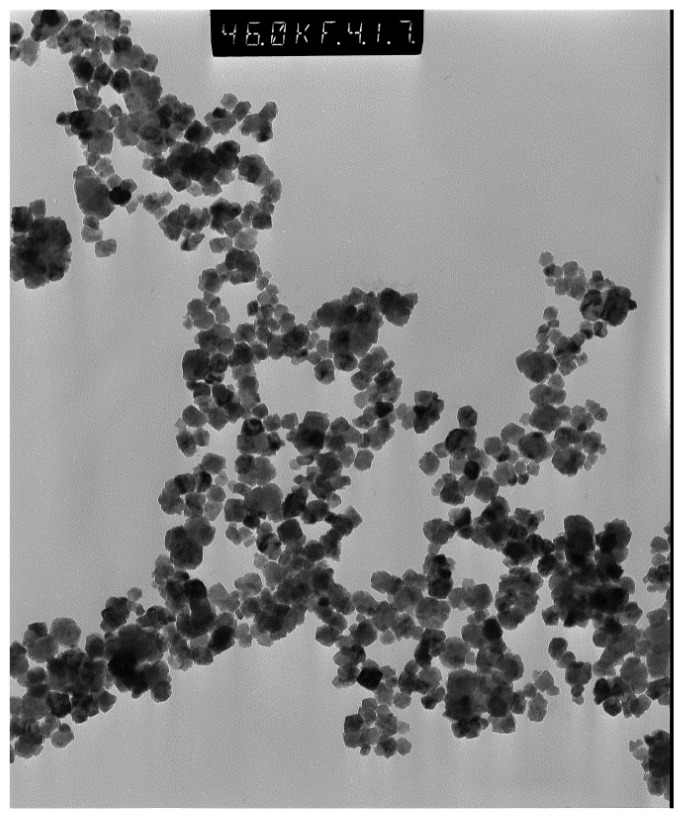
TEM of nanoparticles showing moderate clustering of particles. Size definition becomes more difficult due to agglomeration.

**Figure 4:**
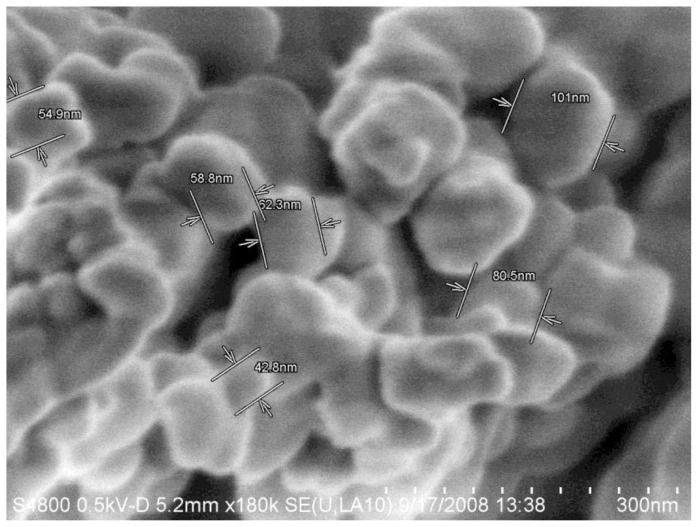
Severe degree of agglomeration of nanoparticles that renders definition of particle morphology and size very difficult. Quantitative analysis in MRI relies on measurement of nanoparticle size and magnetic moment.

Agglomeration of NP will have a direct effect on any measurement performed on NP in order to extract quantitative parameters such as particle size and magnetic moment. Examples of different degrees of agglomeration are shown in Figures 2–4. Severe agglomeration shown in Figure 4 renders the definition of size and shape of the particles almost impossible. All particles shown in Figures 2–4 are Gd-substituted MnZn-ferrites with particles diameter less than 80 nm.

By adjusting the core size, coating thickness, surface chemistry, and targeting ligands, the nanoprobes can be tailored to target specific organs, cells or even molecular markers of different diseases *in vivo* [90,102]. For MRI application as CA coating type and thickness will have a direct effect on relaxivity as will be discussed later.

### 4.4. Biomedical Applications of Magnetic Nanoparticles

In this work we will review, with different emphasis, a few biomedical applications in both therapeutic and diagnostic areas. For example, the use of MNP as MRI contrast agents will be covered in more detail than other applications (e.g., hyperthermia and drug delivery) because many of the particle physical properties discussed earlier in general terms (e.g., surface and agglomeration effects) can be clearly related to MRI. Other applications, such as Magnetic Particle Imaging (MPI) and the development of new nanoparticle-based biosensors are covered elsewhere (see reviews e.g., [128,129]).

#### 4.4.1. Magnetic Hyperthermia

Magnetic hyperthermia (MH) employs MNP as heat sources [130] to raise tissue temperature to ~43 °C at which tumour cells are known to be more sensitive to heat than healthy cells [131]. The synergetic effects of hyperthermia combined with other treatment modalities such as chemotherapy or radiotherapy are currently under extensive investigation. Low concentration of oxygen and nutrients coupled with low pH tend to make cells heat sensitive. Tumours have a tortuous pattern of vessels feeding them and they are unable to dissipate heat as other vessels feeding normal cells do. Above 41 °C heat pushes cancer cells towards acidosis decreased cellular pH) which decreases the cell’s viability and transplant ability, and alters the functions of many enzymatic and structural proteins that affect cell growth and differentiation possibly leading to apoptosis [132]. There have been many studies describing different types of magnetic materials used in the synthesis of HT MNP, different magnetic field strengths, alternating field frequencies and exposure times, and perhaps more importantly a variety of particle delivery and localization techniques (e.g., direct interstitial injection or intravenous delivery in the form of a magnetic fluid [133]). Preferential delivery of the HT particles into the target or tumour site is followed by applying an AC magnetic field of the appropriate frequency that causes the particles to dissipate heat by conduction into the immediately surrounding tissue by mechanisms discussed above [130,134–137]. More details about field frequencies, periods of exposure, *etc.* can be found in review articles [130,137]. The magnetic property of Curie temperature can be utilized to overcome the problems of overheating. If the MNP possesses a Curie temperature ~43 °C, then these particles will lose their strong magnetic properties if heated above this critical temperature and become paramagnetic. This means that absorption of the AC magnetic field will cease even if the field is kept on for longer periods to make sure that all particles, even those at larger tissue depths, have attained the proper temperature. Tailoring the synthesis of the MNP in order to achieve the required therapy temperature becomes important and many research groups have resorted to adding different elements e.g., Gd to iron oxides [72].

The localization of the MNP is obviously critical to the success of hyperthermia as the objective is to spare the surrounding healthy tissue from excessive heat. The implementation of an imaging modality to monitor the delivery and later the dispersion and clearance of the MNP can play an important role in the planning, execution, and evaluating the progress of the study. Among the first clinical studies of treating brain tumours using MH is the one investigating recurrent glioblastoma multiforme via a combination of radiotherapy and MH using aminosaline coated iron oxide nanoparticles (size ~15 nm). MRI was used in selecting the injection site while CT imaging was used in the follow up [138]. More complete evaluation of the clinical outcomes is to be assessed with a larger group of patients. The same group has also began clinical studies on prostate cancer [139,140]. The prospect of combining MH with drug delivery through the use of the same MNP *i.e.*, loaded with drugs, is also receiving considerable attention.

#### 4.4.2. Drug and Gene Delivery

Coated MNP or an agglomeration of them can be functionalized by attaching various molecules such as carboxyl groups, biotin, *etc.* as detailed above [141,142]. These molecules serve to couple various therapeutic drugs or target antibodies to the MNP complex. These magnetic carriers can be used to target specific sites or organs in the body for tumour therapy or gene delivery [143,144]. The drug-loaded carrier is intravenously or intra-arterially into the circulatory system in the form of a biocompatible magnetic fluid. High gradient magnetic field is used to target and concentrate the magnetic carrier at a specific site. The drug can then be released either passively (*i.e*., due to the degradation of the carrier or actively through the application of an actuation in the form of a magnetic pulse or heat. It should be noted that the natural environment of the site can play a role in releasing the drug through various conditions such as pH, osmolality, *etc.* [145]. Obviously targeting the carrier is achieved through a magnetic force exerted through the interaction of the magnetic dipole moment of the MNP and with the applied magnetic field gradient [130]. The success of the targeting and delivery process will depend on a complex interaction of many variables such as the properties of the applied magnetic field and MNP including drug-particle binding, hydrodynamic conditions, MNP concentration, the injection method, and target site location and depth. For example, most available fields are only able to steer the MNP against diffusion and bulk blood flow velocities found in living systems over a distance of few centimeters [146]. Further limitations include the possibility of embolization of the blood vessels due to the accumulation of MNP, and also the fact that once the drug is released it is no longer attached to the magnetic field. Recent efforts have basically concentrated on overcoming these limitations in order to apply these techniques in clinical studies. For example, the use of magnetic needles and meshes have been tested in order to create gradients of magnetic fields of sufficient intensity to overcome hydrodynamic forces and therefore improve targeting [147].

The use of targeting radionuclide via magnetic carriers was investigated to overcome the limitation caused by drug release from the carrier. The radionuclide is actually active throughout the treatment period and does not have to be taken up by the tumour cells. The efficacy of this method was tested on both animal and cell culture studies [148].

A significant development in the area of biomedical applications of MNP has been their use in gene delivery and hence gene therapy. A viral vector carrying the appropriate gene is attached to the coating of the MNP and when approaching the target site gene transfection and expression can ensue and therefore rectifying genetic disorders [149]. Attractive targets for gene therapy include the epithelial surfaces of the lungs and the gastrointestinal tract and endothelial cells lining the blood vessels [150]. Magnetic transfection (or magnetofection) has also aimed at expanding into non-viral transfection of DNA, siRNA, and other biomolecules [151,152] or indeed in studying specific genes involved in disease pathways [153]. Other mechanisms aimed at enhancing the transfection efficacy and the particle-gene endocytosis includes the simultaneous application of ultrasound and magnetic fields [154] or inducing mechanical oscillations for the *in vivo* sample in the direction lateral to the applied magnetic field. The development of *in vivo* gene delivery methods remain at the development stage. Published results on a group of 20 cats with feline fibrosarcomas [155] shows that transfection is well tolerated and that half the group was recurrent-free after one year.

Novel biomedical applications aimed at studying cellular function, mechanical and rheological cell properties, and molecular signal pathways have recently emerged relying on magnetic actuation of cellular components and membranes. Similar physical principles to those discussed above apply where magnetic forces and torques are generated through the application of magnetic fields and their gradients to MNP attached to the cellular components concerned. Pulling or twisting the MNP, via the applied field, can actuate and control specific cellular responses such as activating and deactivating ion channels [156]. An early application of magnetic actuation was the development of magnetic twisting cytometry [157,158] while research work is continuing to address *in vivo* issues such as those in the areas of tissue engineering and regenerative medicine. These areas have been covered in an excellent review by Pankhurst *et al*. [128].

#### 4.4.3. MNP as MRI Contrast Agents (CA)

MRI offers distinct characteristics as an imaging modality in terms of excellent intrinsic soft tissue contrast and safe levels of radiation. Recent advances in hardware technologies [159] have meant that scan times with sub-millimeter resolution can be achieved within 10 ms opening new fields for implementing MRI. Tissue contrast can be further enhanced by the administration of extraneous CA which shortens the longitudinal (*T1*) and transverse (*T2*) relaxation times. Elements possessing unpaired electrons, providing the magnetic moment (*μ*) necessary for dipole-dipole interaction, were first used to enhance protons’ relaxation. Although it was manganese that was used first it was soon superseded by gadolinium with its relatively large magnetic moment due to the seven unpaired electrons. Due to its strong activity Gd was chelated by large molecules shielding it from direct contact with water protons. The increased molecule activity affects its mobility and the time the protons spend in the vicinity of the large Gd magnetic moment. These factors will constitute important elements that govern the inner-sphere relaxation theory used to explain T1 and T2 data in the presence of low concentrations of these paramagnetic CA. Electronic spin relaxation and the relative motion of water protons around the paramagnetic complexes, each with its own characteristic time constant, provides the necessary fluctuations for the dipole-dipole interactions as will be discussed later in more details.

The efficiency of CA can be enhanced by using materials that have larger magnetic moments such as ferromagnetic particles, mainly iron oxides, or indeed ferrites substituted with elements such as Gd, Mn, Zn, *etc.* As explained before these materials behave like giant paramagnetic spins when their volume is reduced to the nanoscale. The relaxation theory of protons in the presence of superparamagnetic NP was developed by modifying the theory of paramagnetic CA taking into account important differences between the two media. These include the size of the magnetic moment and its dependence on the applied magnetic field, the presence of magnetic anisotropy, the particle size and its size distribution, and the particle’s surface/shell nature which strongly modifies the hydrodynamic and magnetic properties of the NP. Due to their high T2 relaxivity (relaxation rate per concentration of MNPs) colloidal suspensions of MNP have been considered for use as MRI CA [160–166].

##### 4.4.3.1. Proton’s Relaxation Due to Paramagnetic Complexes

The use of Gd-chelated complexes to enhance protons’ relaxation is explained by the inner- and outer-sphere relaxation theory as reviewed by others [167,168]. Protons experience electronic spin fluctuations through both dipolar and scalar interactions when they are within close distance of the unpaired Gd electrons, *i.e.*, within the first hydration sphere. These fluctuations, characterized by the electronic longitudinal and transverse correlation times (*τ_S_*_1_ and *τ_S_*_2_), are modulated by the tumbling motion of the protons around the paramagnetic complex (characterized by the rotational correlation time *τ_R_*) and by the residence time (*τ_M_*) of the protons within the first hydration sphere. These correlation times are combined together in the well-known Solomon-Bloembergen theory to describe the inner-sphere relaxation contribution to paramagnetic relaxation [167].

Water molecules wondering farther away from the first hydration sphere still experience dipolar interactions with a characteristic distance “*d*” of closest approach equal or greater than just the radius of the CA complex. The random diffusive motion of the water molecules in the local magnetic field gradients generated by the paramagnetic complex modulate the dipolar interactions with a characteristic time (*τ_D_*) given by the equation [169,170]

(5) $$\tau_{D}=\frac{d^{2}}{D}$$

*D* is the relative diffusion coefficient of the proton and the paramagnetic complex. The outer-sphere contribution to relaxation was described by the Freed equations [170,171].

##### 4.4.3.2. Proton’s Relaxation Due to MNP

The magnetic moment associated with ferro- or super-paramagnetic nanoparticles is much bigger than that associated with paramagnetic atoms. It is hoped that the same effect on relaxation can be produced by a smaller mass of CA material, and hence improve efficiency and reduce the amount of chemicals administered into humans. For superparamagnetic NP the outer-sphere relaxation contribution dominates over the inner-sphere one. Unlike paramagnetic centres, the modulation of the dipolar interactions is provided by Néel’s relaxation of the MNP instead of the electronic relaxation) characterized by the correlation time *τ_N_*; and by the Brownian relaxation characterized by the correlation time *τ_D_* where protons diffuse through non-fluctuating magnetic field gradients created by the mean crystal moment [172–174]. The latter process depends on the particle size, the solvent viscosity and temperature while the former process, as seen before, depends on the anisotropy energy of the particle which increases exponentially with the particle volume. For very small particles (ultrasmall superparamagnetic iron oxide particles—USPIO radius ~5 nm) *E_a_* is very small and thermal energy prevents the crystal’s magnetic moment from locking into the easy (anisotropy) axis.

Earlier studies considered that SPM behaves like a paramagnetic super moment in an isotropic environment, *i.e*., with zero anisotropy [175–177]. The model successfully explained the experimental results for large SPIO crystals (radius ~15 nm) at high fields but failed to agree with relaxation results for smaller particles (USPIO ~5 nm) especially in the low field range where dispersion in the longitudinal NMRD curves occurs [178]. This is characterized by an inflection point where *E τ_C_~ ħ*, where *E*, the total energy, is the sum of the Zeeman (*E_Z_*) and the anisotropy (*E_a_*) energies, and *τ_C_* is the correlation time for the energy fluctuations. For small particles, and hence small *E_a_*, the equality is fulfilled and dispersion is observed. Not so for large particles with *E τ_C_* » *ħ*. Further proof of the role of anisotropy energy was provided by Roch *et al*. [179] when *E_a_* was increased for very small particles by doping with Co (which is known to have high anisotropy) leading to dispersion removal.

Magnetic anisotropy was first taken into account [180] by assuming an infinite *E_a_* with SPM giant moment being locked along one of the easy axes orientations (with no precession) but only Néel’s flipping. This obviously corresponds to the second limit of *E_a_*, *i.e*., infinite *E_a_* instead of *E_a_* zero as discussed before. A quantitative treatment of *E_a_* was successfully implemented [178] for the simple case of uniaxial anisotropy when *E_a_* was treated as a quantitative parameter in the model. A simple, but unrealistic, simulation was considered where both axes of anisotropy and external field are aligned together (*i.e*., situation obtained when applied field is very large) is considered and analytic expressions for 1/T1 and 1/T2 were obtained. The more realistic case where the two axes are separated by an angle was treated and longitudinal relaxivity plots were obtained using a high value for the nanoparticle spin. A linear combination of the two models would be used to fit relaxometry data for NP of both small and large size.

For the high magnetic fields used in MRI Curie relaxation dominates where the NP magnet is locked along B_0_ direction and the relaxation rates are then given by (under certain conditions—see next section) the following equations (similar to Equations (22–24) in [181]; Equations (20) and (21) ignoring Freed’s contribution in [178]; Equations (2) and (3) in [182]).

(6) $$\frac{1}{T_{1}}=\frac{32\pi }{135000}\frac{N_{A}[M]}{RD}\;{\left(\frac{\gamma \mu_{0}}{4\pi }\right)}^{2}\;\left\{\frac{3}{2}J_{A}(\sqrt{2\omega_{I}\tau_{D}})\right\}<\mu_{Z}{>}^{2}$$

(7) $$\frac{1}{T_{2}}=\frac{32\pi }{135000}\frac{N_{A}[M]}{RD}\;{\left(\frac{\gamma \mu_{0}}{4\pi }\right)}^{2}\;\left\{\frac{3}{2}J_{A}(\sqrt{2\omega_{I}\tau_{D}})+J_{A}(0)\right\}<\mu_{Z}{>}^{2}$$

*ω_I_* is the proton’s Larmor frequency, *μ_Z_* is the magnetic moment component along the applied field, and *J_A_* is Ayant’s density spectral function given by

(8) $$J_{A}(z)=\frac{1+5z/8+5z^{2}/8}{1+z+z^{2}+z^{3}/6+4z^{4}/81+z^{5}/81+z^{6}/648}$$

For high fields when *ω_I_* becomes very large (*J_A_*(*z*)→ zero) and using the volumetric particle fraction *f* instead of the molar concentration [*M*] (moles/liter) the relaxation rates can be written as

(9) $$1/T_{1}\to zero$$

(10) $$1/T_{2}=16\;f{\left(\Delta \omega \right)}^{2}\tau_{D}$$

Δ*ω* is the r.m.s. Larmor frequency experienced by the proton at the surface of the particle of radius R and is given by [183].

(11) $$\Delta \omega =\mu_{0}M\;\gamma /3$$

**Figure 5:**
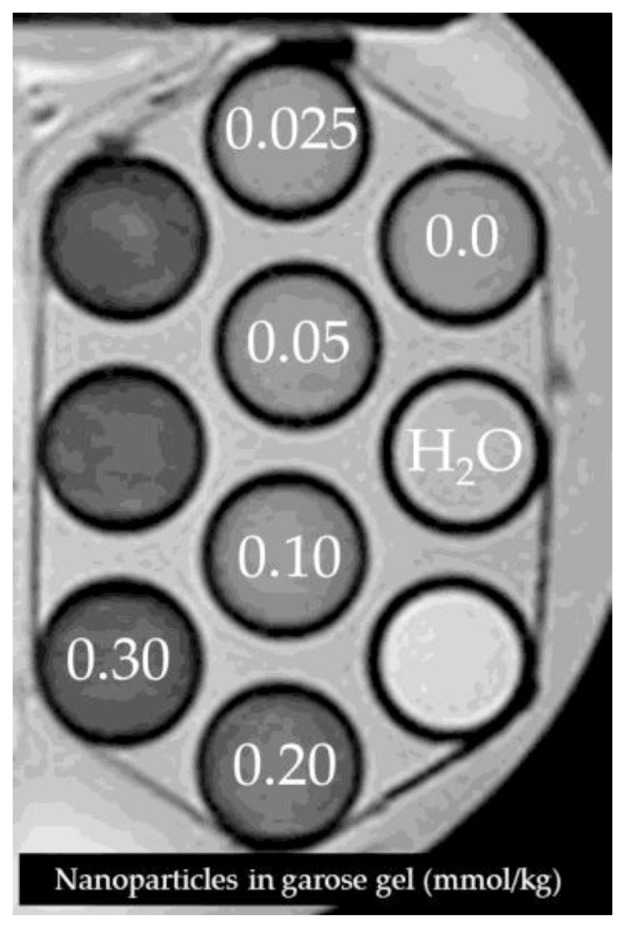
Different concentrations (mM per kg gel) of uncoated Mn_0.5_Zn_0.5_Gd_0.02_Fe_1.98_O_3_ nanoparticles suspended in agarose gel contained in glass tubes surrounded by a water bath [185].

**Figure 6:**
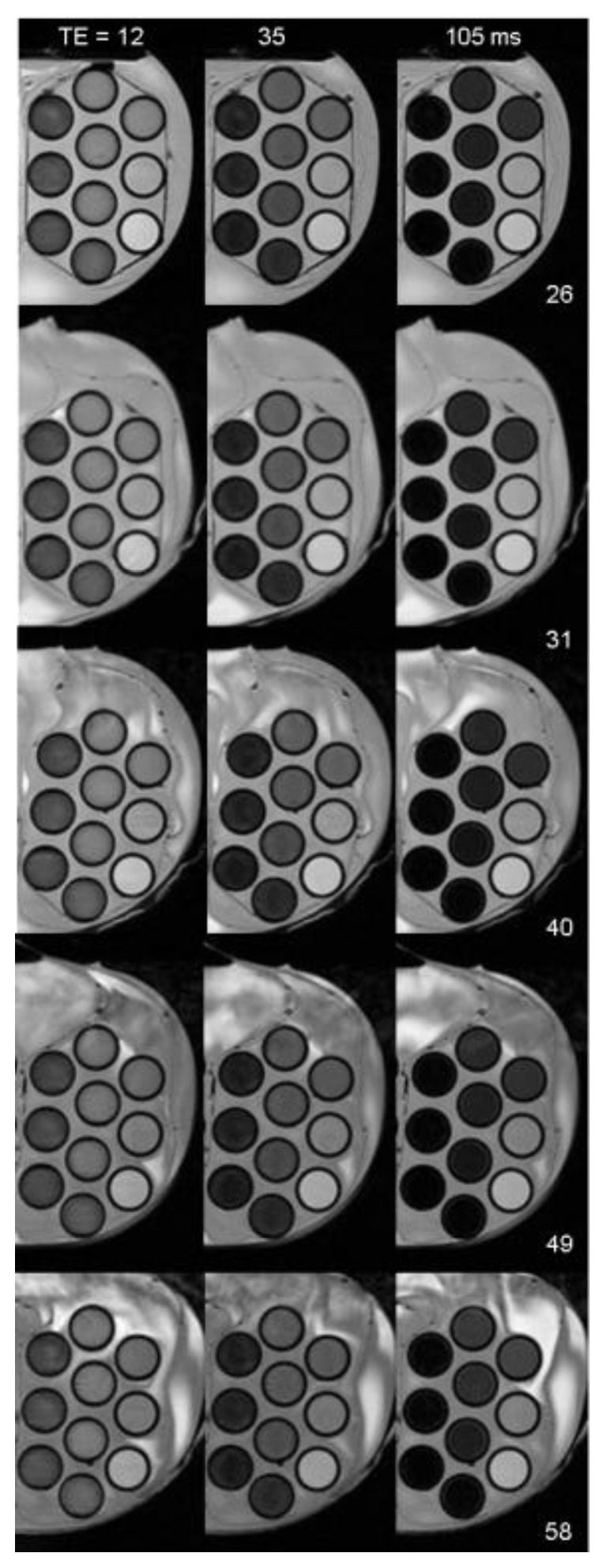
Spin-echo images at 1.5T at different echo times and temperatures. The images show larger signal decay due to higher nanoparticles concentrations, longer echoes, and lower temperatures. In order to understand the signal weighting by *T1* and *T2* relaxation times, quantitative measurement of many nanoparticle-specific parameters are needed such as particle size and the degree of agglomeration. Any possible variation of these parameters (and the magnetic moment) with temperature must also be taken into account.

**Figure 7:**
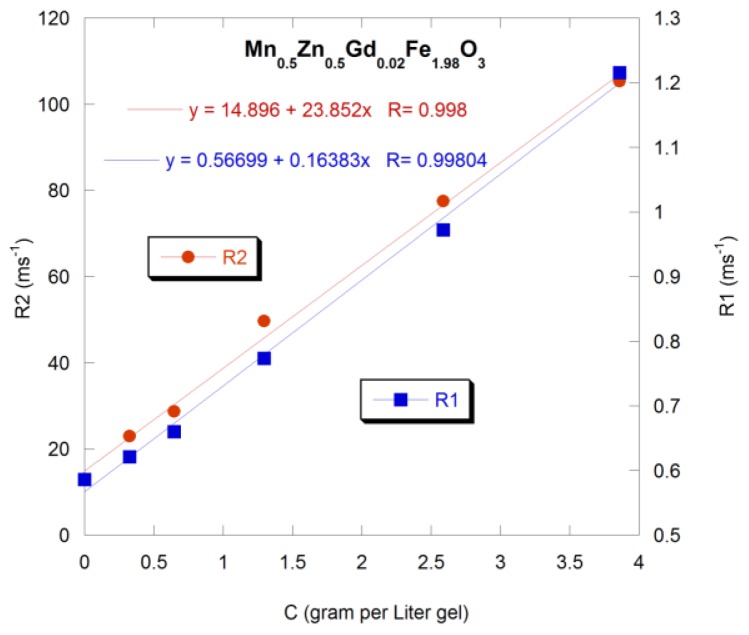
Linear relationship between the relaxation rates and nanoparticle concentration is demonstrated. The slope of the curves defines relaxivity.

A more correct formula include should a factor $\sqrt{4/5}$ that accounts for averaging the dipole magnetic field over a spherical surface [184]. Δ*ω* depends only on the material magnetization and not the size of the particle, for example, magnetite has Δ*ω* = 3.48×10^7^ rads/s. The magnetic frequency can of course be represented in terms of the magnitude of the component of the equatorial magnetic field (*B_eq_*) parallel to the external field *B_o_* using the Larmor relationship. Magnetite’s frequency will then correspond to an equatorial magnetic field *B_eq_* = 1.3 kG. Figures 5 and 6 show an example of the different signal intensity weighting produced by a range of nanoparticle concentration and temperature in a spin-echo experiment. A more quantitative measurement of the longitudinal and transverse relaxation rates *R1*=1/*T1* and *R2*=1/*T2*, respectively, are shown in Figure 7 at 40 °C. Linear relationship of both relaxation rates is demonstrated at the concentrations shown.

##### 4.4.3.3. Frequency Scales and Motional Regimes

Implicit in the above formula (Equations (6), (7) and (10)) is that many encounters of the proton with a nanoparticle are required for it to relax significantly; or equivalently, the motion of the proton has to be very fast in order to experience and average the magnetic fields of many nanoparticles [183]. The water proton is neither confined to the magnetic field of one paramagnetic ion complex nor is completely relaxed by diffusing around one large magnetic nanoparticle [175]. This process defines the dynamic frequency scale or diffusional rate $1/\tau_{D}=\frac{D}{d^{2}}$ (notice the use of “*d*”, the distance of closest approach, or “*R*”, the radius of the nanoparticle by many researchers) defined before in Equation (4). Obviously, the strength of the magnetic dephasing centre has to be taken into account and this is characterized by the magnetic frequency scale which represents the efficiency by which the MNP relaxes a nearby proton as defined above in Equation (10)[186].

The choice of the theoretical model of *T2* relaxation depends on both the size of the particles (*i.e*., mobility) and the magnitude of the magnetic moment (*i.e*., the dephasing effect due to field inhomogeneity) through the product parameter Δ*ω τ_D_*. When the diffusional motion is very fast such that one MNP is unable to completely relax the protons the condition for the motional averaged regime (MAR) is fulfilled 1 /*τ_D_* » Δ*ω* and Equation (10) represents the transverse relaxation rate.

The 1/T2 relaxation rate continues to increase with increasing particle size until reaching an asymptotic limit in 1/T2 (both 1/T2 and 1/T2^*^ are equal and independent of echo time *τ_CP_* where *τ_CP_* is half the echo time for a single (Hahn) spin-echo or half the interval between successive 180° RF pulses in a CPMG sequence) when further particle size increase causes the breakdown of the MAR condition. The limited spins mobility leads to the saturation of 1/T2^*^ value with further particle size increases as given by the static dephasing regime (SDR) where the protons appear less mobile, or static [184,187,188]. Therefore, when Δ*ω τ_D_* » 1, spins relax in the magnetic field gradients created by nearby weakly or strongly magnetized particles and the diffusion coefficient becomes irrelevant. The expressions for 1/*T2* in the SDR regime is given by

(12) $$1/T^{2}=\frac{2\pi }{\sqrt{27}}f\;\Delta \omega$$

For large particles, or agglomeration of particles [189], where the refocusing RF pulses are not effective and the diffusion time *τ_D_* is larger than a characteristic time *τ_L_* given by

(13) $$\tau_{L}=(1.49/\Delta \omega )\;x^{1/3}\;{\left(1.52+f\;x\right)}^{5/3}$$

*x* = Δ*ω τ_CP_*, the theoretical model for *T2*, known as the echo limited regime (ELR), applies and is given by the following Equation (14)

(14) $$1/T2^{ELR}=1.8\;f\;x^{1/3}\;{\left(1.52+f\;x\right)}^{5/3}/\tau_{D}$$

Many researchers have synthesized MRI CA using iron metals [122,190], iron oxides [191] and ferrites substituted by other elements such as Zn, Mn, and Gd [185,192,193], or indeed CA that are based on Gd [194] or gold [195]. Natural magnetized particles were also examined (red blood cell suspensions [196,197] and samples of liver or ferritin [198]) to demonstrate and expand the different relaxation models mentioned above. Different environments, particle sizes, coating types, *etc.* were used under both spin- and gradient-echo conditions. Some studies have attempted to present a quantitative theoretical description [199] to the relaxation motional regime such as weakly [200] or strongly [201] magnetized systems in which the spatial distribution of the magnetic fields was characterized by correlation functions. Another study presented an empirical approach [202] to model the different motional regimes. Monte Carlo numerical simulation was used by other researchers to study *T2* relaxation [186,203,204] for both spherical and cylindrical particle geometry.

**Figure 8:**
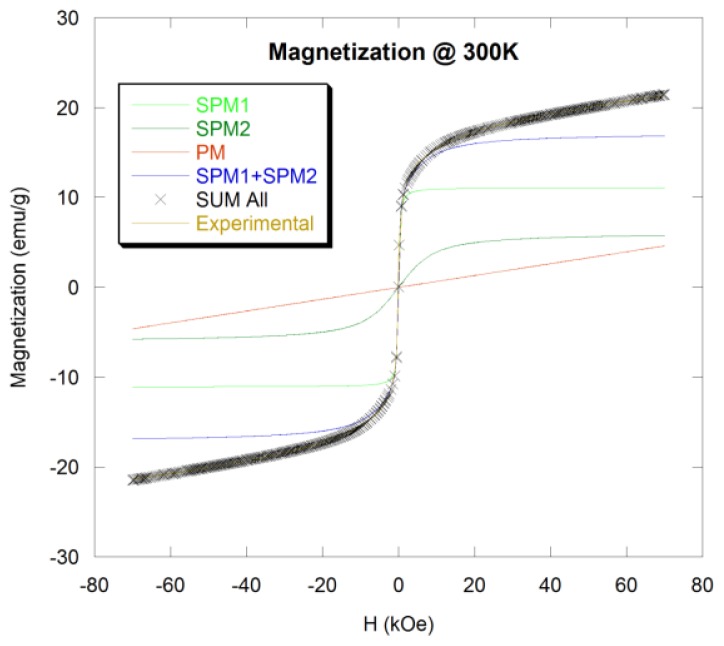
Magnetization curves for Gd-substituted MnZn-ferrite nanoparticles where saturation is not achieved even at very large applied field. Two superparamagnetic and one paramagnetic component are required for good fitting. These extra components can be explained in terms of isolated spins or dead phases (see text).

The role of PEG coating was shown to generally reduce relaxivity [126,205] due to increasing the water protons’ distance of closest approach *i.e*., creating an exclusion zone for the protons from the MNP magnetic moment) and reduced diffusion coefficient. However, increased T1 relaxivity was demonstrated in [126] explained by longer proton’s residence time associated with the reduced diffusion coefficient. While all studies agree that coating increases the hydrodynamic radius of the NP, difficulties still exist in determining the NP size since different physical and magnetic properties are related to NP size in different manners [206]. For example, the physical radius of the NP determining its mobility and diffusion constant may be smaller than the “magnetic size” determining the effectiveness of the NP’s magnetic moment in dephasing the proton. Further difficulties ensue when clustering or agglomeration of nanoparticles exists emphasizing the importance of determining the particle radius as demonstrated by many studies such as those mentioned in [189,205,207]. A more detailed study [208] of the different degrees of agglomeration induced by many types of coating concluded that clustering was the more dominant factor in changing relaxivity over diffusion and exclusion zone. Furthermore, the existence of particle size distribution *i.e*., a polydisperse sample rather than a monodispersed one, is important when trying to fit the various models of relaxation enhancement [209,210]. Particle size distribution, in addition to the existence of multi-magnetization phases, play an important role in accurately determining the particle’s magnetic moment [211]. More than one superparamagnetic magnetic moment component may be needed to explain the magnetization curves in some samples as clearly demonstrated in Figure 8. The isolation of phases during synthesis or the existence of a dead shell layer (e.g., paramagnetic) are necessary to explain the widely reported lack of saturation of the magnetization curve even at very large applied fields [211]. The effect of temperature on relaxivity through a variety of factors such as magnetic moment, clustering and diffusion coefficient was studied in [185] where the implementation of MNP in hyperthermia is considered.

## 5. Conclusions

The aim of this review was to introduce few attributes of magnetic nanoparticles and important physical properties that so often seem confusing for new researchers from other disciplines. In particular, we discuss surface effects (perhaps the single most important property of MNP) and anisotropy due to their importance not only from the fundamental science point of view but also because their understanding is pivotal to the successful implementation of MNP in many biomedicine applications such as MRI and magnetic hyperthermia.

Coating and agglomeration and their subsequent effect on defining size, size distribution, and effective magnetic moment, were discussed and many studies are discussed to highlight practical difficulties that are associated with research. A variety of coating materials (magnetic or non-magnetic with different thicknesses, composition, *etc.*) are currently being studied with direct effect on the functionalization and toxicity of nanoparticles and therefore their applications in biomedicine. These points help to understand the reasons why nanoscale particles have different properties from the bulk scale and why their implementation is successful in such areas as MRI CA which is discussed in more detail in this article.
